# Supplementary material for: Iterative improvement in the automatic modular design of robot swarms
Source: PeerJ Comput Sci. 2020 Dec 7;6:e322. doi: 10.7717/peerj-cs.322 (PMC7924708; doi:10.7717/peerj-cs.322)
Supplement: Supplemental Information 3 [file peerj-cs-06-322-s003.zip › argos3/doc/api/standalone/a00409_source.html]

ARGoS: core/wrappers/lua/lua\_controller.h Source File


- Main Page
- Related Pages
- Namespaces
- Classes
- Files

- File List
- File Members

# core/wrappers/lua/lua\_controller.h

Go to the documentation of this file.

```
00001 
00007 #include <argos3/core/control_interface/ci_controller.h>
00008 #include <argos3/core/utility/math/rng.h>
00009 
00010 extern "C" {
00011 #include <lua.h>
00012 }
00013 
00014 namespace argos {
00015 
00016    class CLuaController : public CCI_Controller {
00017 
00018    public:
00019 
00020       CLuaController();
00021 
00022       virtual ~CLuaController();
00023 
00024       virtual void Init(TConfigurationNode& t_tree);
00025 
00026       virtual void ControlStep();
00027 
00028       virtual void Reset();
00029 
00030       virtual void Destroy();
00031 
00032       inline lua_State* GetLuaState() {
00033          return m_ptLuaState;
00034       }
00035 
00036       virtual void SetLuaScript(const std::string& str_script,
00037                                 TConfigurationNode& t_tree);
00038 
00039       virtual void SetLuaScript(const std::string& str_script);
00040 
00041       virtual void CreateLuaState();
00042 
00043       virtual void SensorReadingsToLuaState();
00044 
00045       virtual void ParametersToLuaState(TConfigurationNode& t_tree);
00046 
00047       inline bool IsOK() const {
00048          return m_bIsOK;
00049       }
00050 
00051       std::string GetErrorMessage();
00052 
00053    private:
00054 
00055       lua_State* m_ptLuaState;
00056       std::string m_strScriptFileName;
00057       bool m_bScriptActive;
00058       bool m_bIsOK;
00059       CRandom::CRNG* m_pcRNG;
00060 
00061    };
00062 
00063 }
```

---

Generated on 10 Jul 2018 for ARGoS by 
 1.6.1
